# Serious adverse events, readmission, and mortality after shoulder replacement due to fracture, osteoarthritis, and other indications: a population-based comparison with the general population

**DOI:** 10.2340/17453674.2025.44796

**Published:** 2025-10-03

**Authors:** Josefine Meyer LARSEN, Martin Gade STISEN, Pia Kjær KRISTENSEN, Antti P LAUNONEN, Theis Muncholm THILLEMANN, Inger MECHLENBURG

**Affiliations:** 1 Department of Clinical Medicine, Aarhus University, Aarhus N, DDenmark; 2 Department of Orthopaedic Surgery, Aarhus University Hospital, Aarhus N, Denmark; 3 Department of Orthopaedic Surgery, Tampere University Hospital, Tampere Finland; 4 Department of Public Health, Aarhus University, Aarhus, Denmark; 5 VIA University College, Research Center for Activity and Prevention, Aarhus N, Denmark

## Abstract

**Background and purpose:**

Patients treated with shoulder arthroplasty may risk serious adverse events (SAEs), readmission, and death; however, the literature is inconsistent. Therefore, we aimed to compare the incidence rates of SAEs, readmissions, and mortality at 30 and 90 days following shoulder replacement with those of a matched cohort.

**Methods:**

Danish databases were used to include patients treated with a primary shoulder replacement due to fracture, osteoarthritis, cuff tear arthropathy, and other (2006–2021). The shoulder patients were compared (1:10) to a matched cohort from the general population. Incidence rates (IR) and incidence rate ratios (IRR) were calculated and adjusted for age, sex, and comorbidity.

**Results:**

The 30-day IR of SAEs was 73.5 for shoulder patients and 14.8 for the matched cohort. The IRR of SAEs was higher for all patient groups compared with the matched cohort and varied between indications for surgery (IRR 3.1–5.9) and remained higher at 90 days (IRR 1.6–3.5). The IR of readmission was 234 per 100,000 person-days at 30 days. The 30-day IR of mortality was 20.2 per 100,000 person-days for shoulder patients and 9.4 per 100,000 person-days for the matched cohort. Compared with the matched cohort the 30-day IRR of mortality was 2.0, with fracture patients having the highest risk of mortality (IRR of 3.5).

**Conclusion:**

At 30 and 90 days after surgery, shoulder patients, regardless of surgical indications, had higher rates of SAEs than the matched cohort. The mortality rate was higher for shoulder patients and highest for fracture patients. This information should be included in the shared decision-making process before undergoing shoulder replacement.

Shoulder replacement is used to treat glenohumeral osteoarthritis, proximal humerus fractures, and rotator cuff tear arthropathy, among other conditions [1]. The most common procedures are total anatomical shoulder replacement, reverse total shoulder replacement, and hemiarthroplasty [1].

When deciding on a treatment, shared decision-making should be used. This evidence-based approach must explain potential risks, benefits, and alternatives to surgery [2]. While revision rates are well documented, evidence on serious adverse events (SAEs), readmission, and short-term mortality is inconsistent [3-10].

Existing studies often rely on small and selected cohorts and lack comparisons with background population risks. No previous study has used matched general population data to contextualize short-term risks following shoulder replacement. We therefore conducted a nationwide cohort study using linked Danish health registries to estimate the incidence of SAEs, hospital readmission, and mortality, at 30 and 90 days following shoulder replacement. We compared these rates with those of a matched general population cohort.

## Methods

### Study design and setting

This population-based prospective cohort study used routinely collected data from shoulder replacements performed at public and private hospitals in Denmark between January 1, 2006, and December 31, 2021.

The study was reported according to STROBE guidelines.

### Data sources

Data from the Danish Shoulder Arthroplasty Registry were linked to the Danish National Patient Registry, the Danish Civil Registration System, and Statistics Denmark.

The Danish Shoulder Arthroplasty Registry is a national clinical register under the Danish Clinical Quality Program–National Clinical Registries (RKKP) on shoulder replacements performed in Denmark. It includes primary and revision shoulder arthroplasties. All public orthopedic departments and private hospitals in Denmark performing shoulder arthroplasties report to the register. The completeness of the registry was found to be 94.4% in a recent validation study [11].

The Danish National Patient Registry contains information on all somatic patients from hospitals, outpatient clinics, and emergency room visits. Data from the hospitals has been collected since 1977 and the remaining data has been collected since 1995 [12]. Data on SAEs and readmission were derived from this registry.

The Danish Civil Registration System provides every citizen in Denmark with a unique 10-digit personal identification number, which is included in all the Danish medical databases. This allows for individual-level linkage of Danish registers. It is updated daily with information on vital status and migration and allows for complete follow-up of all patients [13]. Information on all-cause mortality was extracted from the Danish Civil Registration System.

The Statistics Denmark database contains data on socioeconomic status and is updated yearly. Cohabiting status, education, family liquid assets, and labor market status were extracted from this database.

### Participants

Participants aged 18 to 100 years with an incident primary shoulder replacement were eligible for this study. Those without Danish residency during the follow-up period or without a Danish social security number were excluded. The shoulder replacement cohort was matched on birth year and sex with a randomly selected cohort (1:10) from the general Danish population. The 1:10 matching ratio was chosen to increase precision. Individuals in the matched cohort had not undergone shoulder replacement or any other shoulder surgeries in the 10 years prior to the index date. The index date for the matched cohort was the surgical date of the matched shoulder patients.

Shoulder patients were categorized by surgical indication: fracture (acute and older fractures and pseudoarthrosis), osteoarthritis (primary and secondary), cuff tear arthropathy (massive rotator cuff tears with or without degenerative changes), and other (avascular necrosis, rheumatoid arthritis, malignancy, and missing indications).

### Outcomes

#### Serious adverse events

At 30 and 90 days after surgery SAEs were examined. SAEs included prolonged hospitalization or new hospitalization due to complications/diagnoses identified using the following ICD-10 codes (International Classification of Diseases, 10th version): pulmonary embolism, myocardial infarction, lower respiratory tract infection, acute kidney injury, urinary tract infection, cerebrovascular events, and sepsis (Table 1 in Appendix).

**Table 1:** Serious adverse events definition and ICD-10 codes

	ICD-10 code
Pulmonary embolism	I26
Myocardial infarction	I21, I22
Lower respiratory tract infection	J12, J13, J14, J15, J16, J18, J22, J86, J440, J851, J690
Acute kidney injury	N17
Urinary tract infection	N10, N300, N308, N309, N390
Cerebrovascular event	I60, I61, I62, I63, I64
Sepsis	A40, A41, B37.7, A32.7, A54.8G, A02.1, A22.7, A26.7, A42.7, A28.2B

#### Readmission

Readmission was defined as any hospitalization lasting 24 hours or more after discharge, classified using ICD-10 codes. Patients without a discharge date for their primary surgery were excluded from the analysis.

For the matched cohort, SAEs were defined as hospital admissions with a diagnosis corresponding to 1 of the specified complications. As these individuals had not undergone surgery, the term “readmission” was not applicable; instead, all relevant hospital contacts were categorized as admissions.

#### All-cause mortality

All-cause mortality occurring within 30 and 90 days and 1 year after surgery were examined.

### Covariates

Covariates included sex, age at index date, Charlson Comorbidity Index, cohabitation status at index date, highest obtained education status, family liquid assets, and labor market status.

Charlson Comorbidity Index was calculated for all patients, encompassing a 10-year period prior to the index date. Charlson Comorbidity Index was defined as low (score 0), medium (score 1–2), and high (score > 2).

Cohabiting was categorized into living alone, cohabiting, and other. Other included households with multiple families.

Education was derived using data from the Population Education Register within Statistics Denmark. Low was defined as none or primary and lower school, medium as vocational education or higher general and preparatory examination, and high as higher education.

Family liquid assets were derived from the Income Statistics Register within Statistics Denmark and were calculated as an average family income and liquid assets from index date and 5 years prior. These were categorized into tertiles of increasing liquid assets.

Labor-market status was derived from Statistics Denmark and was categorized into unemployed, employed, retired, or other/missing.

### Statistics

Descriptive statistics summarized continuous variables as means and categorical variables as proportions. Incidence rates (IRs) were calculated as the number of new cases divided by the product of the population size and time with time units defined as person-days. This calculation was done separately for shoulder patients and the matched cohort for the mortality at 30 days, 90 days, and 1 year, and for SAEs at 30 and 90 days. Readmission rates were calculated only for shoulder patients at 30 and 90 days. For mortality and SAEs, we also calculated an incidence rate ratio (IRR) between shoulder patients and the matched cohort to compare the incidence rates. IRRs were crude and adjusted for sex, age, and Charlson Comorbidity Index. All analyses were done using the statistical software, STATA 18 (StataCorp LLC, College Station, TX, USA).

### Ethics approval, data sharing, funding, and disclosures

According to Danish law, register-based studies do not require approval from an ethics committee. The study was reported to the Danish Data Protection Agency list of research projects (Journal No 1-16-02-387-22).

Access to the data requires permission from the Danish Shoulder Arthroplasty Registry and Statistics Denmark. Information can be found at https://www.rkkp.dk/kvalitetsdatabaser/dansk-skulderalloplastik & https://www.dst.dk/en.

Funding was received from Aarhus University, the Association of Danish Physiotherapists, Dagmar Marshall Foundation, L.F. Foght Foundation, Emil Hertz Foundation, K. A. Rohde Foundation, and Orthopedic Research Foundation Aarhus. The foundations provided financial support and had no role in the planning of the study. The authors declare no conflicts of interest. JML, TMT, and PKK are all members of the steering committee for the Danish Shoulder Arthroplasty Registry. Complete disclosure of interest forms according to ICMJE are available on the article page, doi: 10.2340/17453674.2025.44796

**Figure:**
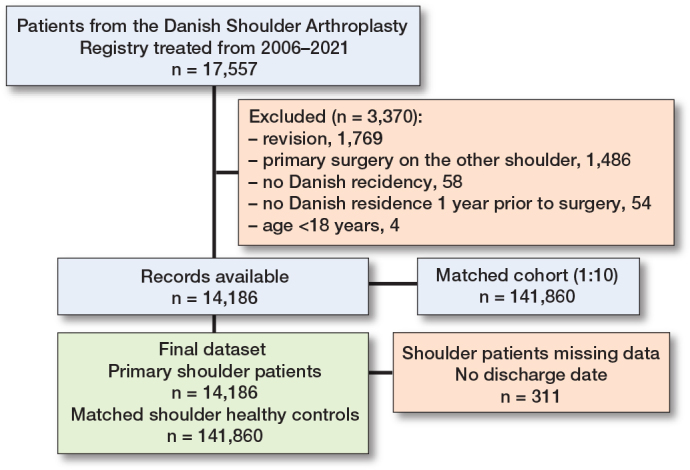
Flowchart of included shoulder patients and matched controls.

## Results

14,186 primary shoulder replacement procedures were matched with a cohort of 141,860 participants (Table 2). The mean age was 70 (standard deviation [SD] 10.8) years, and 68% were female. The 14,186 primary shoulder arthroplasties were performed for the indications: fracture (39%), shoulder osteoarthritis (36%), rotator cuff tear arthropathy (16%), and other (8.6%). The prosthesis types used were hemiarthroplasty (50%), reverse total shoulder replacement (31%), anatomical total shoulder replacement (19%), and unknown prosthesis type (0.3%). 311 patients without a discharge date were excluded from the readmission analysis (Figure).

**Table 2:** Study population and a cohort matched on birth year and sex randomly selected (1:10) from the general Danish population presented as numbers (%) unless otherwise specified

Item	Shoulder patients	Matched cohort
	n = 14,186	n = 141,860
Female sex	9,650 (68)	96,500 (68)
Age, mean (SD)	70 (10.8)	70 (10.8)
Charlson Comorbidity Index		
Low (0)	9,521 (67)	108,269 (76)
Medium (1–2)	3,560 (25)	26,763 (19)
High (> 2)	1,105 (7.8)	6,828 (4.8)
Cohabiting		
Living alone	6,361 (45)	56,874 (40)
Cohabiting	6,957 (49)	75,847 (54)
Other **^a^**	868 (6.1)	9,139 (6.4)
Education **^b^**		
Low	6,013 (42)	54,668 (39)
Medium	5,692 (40)	56,291 (40)
High	2,070 (15)	26,645 (19)
Missing	411 (2.9)	4,256 (3.0)
Family liquid assets or income		
Low tertile	5,776 (41)	46,228 (33)
Medium tertile	4,633 (33)	47,370 (33)
High tertile	3,777 (27)	48,226 (34)
Missing	–	36 (0.03)
Labor market status		
Unemployed	571 (4.0)	2,253 (1.6)
Employed	1,929 (14)	30,442 (25)
Retired	11,571 (82)	107,840 (76)
Other/missing	115 (0.8)	1,325 (0.9)

a Other includes households with multiple families.

b Education: Low is defined as none or primary and lower school. Medium is defined as vocational education or higher general and preparatory examination programs. High is defined as higher education.

### Serious adverse events

The 30-day rate of SAEs was 2.2% for shoulder patients and 0.44% for the matched cohort, with an IR of 73.5 per 100,000 person-days for shoulder patients compared with 14.8 per 100,000 person-days in the matched cohort, corresponding to an IRR of 5.0 (CI 4.3–5.7). Adjusting for age, sex, and Charlson Comorbidity Index yielded similar results. The rate of SAEs varied by surgical indication. Patients with fractures had the highest rate, with an adjusted IRR of 5.9 (CI 5.0–7.1). For patients with osteoarthritis and cuff tear arthropathy, SAEs were higher compared with the matched cohort. Although rates decreased at 90 days, the associations remained unchanged (Table 3).

**Table 3:** Absolute and relative measures of all-cause mortality and serious adverse events for the surgical indications in 14,186 shoulder patients and 141,860 matched cohort participants

Surgical indication	Shoulder patients		Matched cohort		Unadjusted	Adjusted
	n (%)	IR (CI)	n (%)	IR (CI)	IRR (CI)	IRR ^a^ (CI)
30-days mortality						
Total	86 (0.6)	20.3 (16.4–25.0)	401 (0.3)	9.4 (8.6–10.4)	2.2 (1.7–2.7)	2.0 (1.6–2.5)
Fracture	68 (1.2)	41.0 (32.3–52.0)			4.3 (3.4–5.6)	3.5 (2.7–4.6)
OA and CTA	8 (0.2)	3.6 (1.8–7.2)			0.4 (0.2–0.8)	0.4 (0.2–0.8)
Other	10 (0.9)	28.9 (15.6–53.7)			3.1 (1.6–5.7)	2.5 (1.3–4.7)
30-days SAE						
Total	307 (2.2)	73.5 (65.7–82.2)	629 (0.4)	14.8 (13.7–16.0)	5.0 (4.3–5.7)	4.6 (4.0–5.3)
Fracture	166 (3.0)	102.1 (87.7–118.9)			6.9 (5.8–8.2)	5.9 (5.0–7.1)
OA	61 (1.2)	39.8 (31.0–51.2)			2.7 (2.1–3.5)	3.1 (2.4–4.0)
CTA	50 (2.2)	73.5 (55.7–97.0)			5.0 (3.7–6.6)	3.7 (2.8–5.0)
Other	30 (2.6)	87.9 (61.4–125.7)			5.9 (4.1–8.5)	4.8 (3.3–7.0)
90-days mortality						
Total	197 (1.4)	15.6 (13.5–17.9)	1,107 (0.8)	8.7 (8.2–9.2)	1.8 (1.5–2.1)	1.7 (1.4–1.9)
Fracture	146 (2.6)	29.6 (25.1–34.8)			3.4 (2.9–4.0)	2.8 (2.3–3.3)
OA	15 (0.3)	3.2 (2.0–5.4)			0.4 (0.2–0.6)	0.5 (0.3–0.8)
CTA	8 (0.4)	3.9 (1.9–7.7)			0.4 (0.2–0.9)	0.3 (0.2–0.7)
Other	28 (2.4)	27.2 (18.8–39.4)			3.1 (2.2–4.6)	2.7 (1.9–3.9)
90-days SAE						
Total	470 (3.3)	37.9 (34.7–41.5)	1,731 (1.2)	13.7 (13.1–14.3)	2.8 (2.5–3.1)	2.6 (2.3–2.8)
Fracture	264 (4.7)	55.1 (48.8–62.2)			4.0 (3.5–4.6)	3.5 (3.1–4.0)
OA	91 (1.78)	19.9 (16.2–24.4)			1.5 (1.2–1.8)	1.6 (1.3–2.0)
CTA	73 (3.2)	36.1 (28.7–24.4)			2.3 (2.1–3.3)	2.0 (1.6–2.5)
Other	42 (3.6)	41.8 (30.9–56.5)			3.1 (2.2–4.2)	2.6 (1.9–3.5)
1-year mortality						
Total	507 (3.6)	10.0 (9.2–10.9)	4,444 (3.1)	8.7 (8.5–9.0)	1.2 (1.1–1.3)	1.1 (1.0–1.2)
Fracture	341 (6.1)	17.4 (15.7–19.3)			2.0 (1.8–2.2)	1.6 (1.5–1.8)
OA	50 (0.9)	2.7 (2.0–3.5)			0.3 (0.2–0.4)	0.4 (0.3–0.5)
CTA	34 (1.5)	4.1 (2.9–5.7)			0.5 (0.3–0.7)	0.3 (0.2–0.5)
Other	82 (7.1)	20.2 (16.3–25.1)			2.3 (1.9–2.9)	2.1 (1.6–2.6)

CI: 95% confidence interval; CTA: rotator cuff tear arthropathy; IR: incidence rate per 100,000 person-days; IRR: incidence rate ratio per 100,000 person-days. OA: osteoarthritis.

a Adjusted for sex, age, and Charlson comorbidity index.

For all SAEs, the 30-day IRs were higher for the shoulder patients compared with the matched cohort, with similar results when adjusting for age, sex, and Charlson Comorbidity Index. The most common SAE for shoulder patients was lower respiratory tract infection, with an IR of 34.9 per 100,000 person-days compared with 7.0 per 100,000 person-days in the matched cohort (Table 4). The highest rate for specified SAEs compared with the matched cohort was acute kidney injury with an adjusted IRR of 17.1 (CI 9.0–32.2), followed by pulmonary embolism with an adjusted IRR of 10.4 (CI 6.5–16.8). The rates were lower at 90 days, but the associations remained consistent (Table 4).

**Table 4:** Absolute and relative measures of serious adverse events in 14,186 shoulder patients and 141,860 matched cohort participants

SAE by follow up	Shoulder patients		Matched cohort		Unadjusted	Adjusted
	n (%)	IR (CI)	n (%)	IR (CI)	IRR (CI)	IRR^a^ (CI)
Pulmonary embolism						
30 days	39 (0.3)	9.3 (6.8–12.8)	35 (0.02)	0.8 (0.6–1.1)	11.3 (7.2–17.9)	10.4 (6.5–16.8)
90 days	53 (0.4)	4.3 (3.3–5.6)	84 (0.06)	0.7 (0.5–0.8)	6.4 (4.6–9.1)	5.9 (4.1–8.4)
Myocardial infarction						
30 days	17 (0.1)	4.1 (2.5–6.5)	52 (0.04)	1.2 (0.9–1.6)	3.3 (1.9–5.7)	3.2 (1.9–5.7)
90 days	24 (0.2)	1.9 (1.3–2.9)	187 (0.1)	1.5 (1.3–1.7)	1.3 (0.9–2.0)	1.3 (0.8–1.9)
Lower respiratory tract infection						
30 days	146 (1.0)	34.9 (29.7–41.1)	298 (0.2)	7.0 (6.3–7.9)	5.0 (4.1–6.1)	4.6 (3.7–5.6)
90 days	216 (1.5)	17.4 (15.3–19.9)	806 (0.6)	6.4 (6.0–6.8)	2.7 (2.4–3.2)	2.5 (2.1–2.9)
Urinary tract infection						
30 days	25 (0.2)	6.0 (4.0–8.9)	60 (0.04)	1.4 (1.1–1.8)	4.2 (2.7–6.7)	4.0 (2.5–6.5)
90 days	49 (0.4)	4.0 (3.0–5.2)	158 (0.1)	1.2 (1.1–1.5)	3.2 (2.3–4.4)	3.0 (2.2–4.2)
Acute kidney injury						
30 days	31 (0.2)	7.4 (5.2–10.6)	16 (0.01)	0.4 (0.2–0.6)	19.7 (10.8–40.0)	17.1 (9.0–32.2)
90 days	40 (0.3)	3.2 (2.4–4.4)	50 (0.04)	0.4 (0.3–0.5)	8.2 (5.4–12.4)	7.2 (4.8–11.2)
Cerebrovascular event						
30 days	23 (0.2)	5.0 (3.7–8.3)	96 (0.07)	2.3 (1.9–2.8)	2.4 (1.5–3.8)	2.4 (1.5–3.7)
90 days	45 (0.3)	3.6 (2.7–4.9)	279 (0.2)	2.2 (2.0–2.5)	1.6 (1.2–2.6)	1.6 (1.2–2.2)
Sepsis						
30 days	26 (0.2)	6.2 (4.2–9.1)	72 (0.05)	1.7 (1.4–2.1)	3.7 (2.3–5.7)	3.3 (2.1–5.1)
90 days	43 (0.3)	3.5 (2.6–4.7)	167 (0.1)	1.3 (1.1–1.5)	2.6 (1.9–3.7)	2.4 (1.7–3.3)

For abbreviations, see Table 3.

SAEs were most frequent for patients treated for fractures with an IRR of 5.9 (CI 5.0–7.1) compared with the matched cohort. For the majority of SAEs, the highest rates were observed in patients with either fractures or other surgical indications. For pulmonary embolism, the adjusted IRR was highest in patients with osteoarthritis at 30 days, with an IRR of 11.0 (CI 5.8–20.9), and in those with cuff tear arthropathy at 90 days, with an IRR of 6.0 (CI 3.0–11.7) (Tables 5 and 6).

**Table 5:** Absolute and relative measures of 30-day serious adverse events within the surgical indication groups in 14,186 shoulder patients and 141,860 matched cohort participants

SAE and surgical indication ^b^	Shoulder patients		Matched cohort		Unadjusted	Adjusted
	n (%)	IR (CI)	n (%)	IR (CI)	IRR (CI)	IRR^a^ (CI)
Pulmonary embolism			35 (0.02)	0.8 (0.6–1.1)		
Fracture	4 (0.4)	9.2 (5.5–15.3)			11.1 (6.1–20.4)	9.0 (4.8–17.0)
OA	13 (0.3)	8.4 (4.9–14.6)			10.3 (5.4–19.4)	11.0 (5.8–20.9)
CTA	7 (0.3)	10.2 (4.9–21.6)			12.5 (5.5–28.1)	10.2 (4.4–23.6)
Other	4 (0.3)	11.7 (4.4–31.2)			14.1 (5.0–39.9)	8.5 (2.7–26.0)
Lower respiratory tract infection			298 (0.2)	7.0 (6.3–7.9)		
Fracture	76 (1.3)	46.8 (37.3–58.5)			6.6 (5.2–8.6)	5.6 (4.3–7.3)
OA	28 (0.5)	18.3 (12.6–26.5)			2.6 (1.8–3.8)	3.0 (2.0–4.4)
CTA	30 (1.3)	44.1 (30.8–63.0)			6.3 (4.3–9.1)	4.6 (3.1–6.8)
Other	12 (1.0)	35.1 (20.0–61.8)			5.0 (2.8–8.9)	3.7 (2.0–6.7)
Urinary tract infection **^b^**			60 (0.04)	1.4 (1.1–1.8)		
Fracture	14 (0.3)	8.6 (5.1–14.5)			6.1 (3.4–10.8)	5.0 (2.7–9.1)
OA	6 (0.2)	3.9 (1.7–8.7)			2.8 (1.2–6.4)	3.5 (1.5–8.2)
CTA	–	–			–	–
Other	–	–			–	–
Acute kidney injury			16 (0.01)	0.4 (0.2–0.6)		
Fracture	16 (0.3)	9.8 (6.0–16.1)			26.0 (13.0–52.2)	22.8 (10.6–48.8)
OA	4 (0.1)	2.6 (0.9–6.9)			6.9 (2.3–20.7)	6.7 (2.2–20.5)
CTA	6 (0.3)	8.8 (3.9–19.6)			23.3 (9.1–59.7)	15.6 (6.0–40.6)
Other	5 (0.4)	14.6 (6.1–35.1)			38.8 (14.2–106)	17.6 (5.6–55.1)
Sepsis			72 (0.05)	1.7 (1.4–2.1)		
Fracture	13 (0.2)	8.0 (4.6–13.7)			4.7 (2.6–8.5)	4.2 (2.3–7.7)
OA	5 (0.1)	3.3 (1.4–7.8)			1.9 (0.8–4.7)	2.2 (0.9–5.4)
CTA	4 (0.2)	5.8 (2.2–15.7)			3.4 (1.3–9.5)	2.5 (0.9–6.7)
Other	4 (0.4)	11.7 (4.4–31.2)			6.9 (2.5–18.9)	6.4 (2.3–17.6)

For abbreviations, see Table 2.

b There were too few events to allow for data extraction for myocardial infarction, cerebrovascular event, and urinary tract infection on all surgical indication levels.

**Table 6:** Absolute and relative measures of 90-day serious adverse events within the surgical indication groups in 14,186 shoulder patients and 141,860 matched cohort participants

SAE and surgical indication^b^	Shoulder patients		Matched cohort		Unadjusted	Adjusted
	n (%)	IR (CI)	n (%)	IR (CI)	IRR (CI)	IRR^a^ (CI)
Pulmonary embolism			84 (0.06)	0.7 (0.5–0.8)		
Fracture	22 (0.4)	4.5 (3.0–6.9)			6.9 (4.3–11.0)	5.8 (3.6–9.3)
OA	16 (0.3)	3.5 (2.1–5.7)			5.3 (3.1–9.0)	5.7 (3.3–9.7)
CTA	10 (0.4)	4.9 (2.7–9.2)			7.4 (3.8–14.3)	6.0 (3.0–11.7)
Other	5 (0.4)	4.9 (2.0–11.9)			7.5 (3.0–18.5)	5.3 (2.1–13.2)
Lower respiratory tract infection			806 (0.6)	6.4 (6.0–6.8)		
Fracture	119 (2.1)	24.8 (20.8–29.7)			3.9 (3.2–4.7)	3.3 (2.7–4.0)
OA	39 (0.8)	8.5 (6.2–11.6)			1.3 (1.0–1.8)	1.5 (1.1–2.1)
CTA	38 (1.7)	18.7 (13.7–25.8)			2.9 (2.1–4.1)	2.2 (1.5–3.0)
Other	20 (1.7)	19.9 (12.8–30.8)			3.1 (2.0–4.8)	2.4 (1.5–3.7)
Urinary tract infection			158 (0.1)	1.2 (1.1–1.5)		
Fracture	27 (0.5)	5.6 (3.9–8.2)			4.5 (3.0–6.8)	3.7 (2.5–5.6)
OA	11 (0.2)	2.4 (1.3–6.6)			1.9 (1.0–3.5)	2.3 (1.2–4.3)
CTA	6 (0.3)	3.0 (1.3–6.6)			2.3 (1.0–5.3)	1.8 (0.8–4.2)
Other	5 (0.4)	4.9 (2.0–11.9)			3.9 (1.6–9.7)	3.7 (1.5–9.3)
Acute kidney injury			50 (0.04)	0.4 (0.3–0.5)		
Fracture	22 (0.3)	4.5 (3.0–6.9)			11.6 (7.0–19.2)	10.5 (6.2–17.7)
OA	7 (0.1)	1.5 (0.7–3.2)			3.8 (1.8–8.5)	4.1 (1.9–9.2)
CTA	6 (0.3)	3.0 (1.3–6.6)			7.5 (3.2–17.5)	5.4 (2.3–12.6)
Other	5 (0.4)	4.9 (2.0–11.9)			12.5 (5.0–31.6)	8.8 (3.4–22.7)
Sepsis			167 (0.1)	1.3 (1.1–1.5)		
Fracture	23 (0.4)	4.8 (3.1–7.2)			3.6 (2.3–5.6)	3.2 (2.1–5.0)
OA	8 (0.2)	1.7 (0.9–3.5)			1.3 (0.7–2.6)	1.5 (0.7–3.0)
CTA	7 (0.3)	3.4 (1.6–7.3)			2.6 (1.2–5.5)	1.8 (0.8–4.0)
Other	5 (0.4)	4.9 (2.0–11.9)			3.8 (1.5–9.2)	3.3 (1.4–8.3)

For abbreviations, see Tables 3 and 5..

### Readmissions

The 30-day readmission IR for all shoulder patients was 234 per 100,000 person-days. It was highest for the “other” surgical indication, with an IR of 334 per 100,000 person-days, followed closely by fractures with an IR of 318 per 100,000 person-days. At 90 days, the readmission rates were highest for fractures, followed by the “other” indication (Table 7). The most common reason for readmission was “injury, poisoning, and other external causes” (ICD-10: S00-T98) (Table 8).

**Table 7:** Absolute and relative measures of all-cause readmission at 30 and 90 days for the surgical indications for 13,875 shoulder patients

Readmission by surgical indication	No. of events	IR (CI)
Total		
30 days	926	234 (219–249)
90 days	1,536	133 (127–140)
Fracture		
30 days	485	318 (291–348)
90 days	809	185 (173–199)
OA		
30 days	188	128 (111–147)
90 days	336	77 (69–86)
CTA		
30 days	148	227 (193–267)
90 days	228	120 (105–137)
Other		
30 days	105	334 (276–404)
90 days	163	181 (155–210)

For abbreviations, see Tables 3 and 5.

**Table 8:** Reason for all-cause readmission at 30 and 90 days after discharge for 13,875 shoulder patients presented as count (%)

Reason (ICD-10 chapters)		30 days	90 days
A0–B99	(Certain infectious and parasitic diseases)	40 (4.1)	56 (3.5)
C00–D48	(Neoplasms)	26 (2.7)	52 (3.2)
D50–D89	(Diseases of the blood and blood-forming organs and certain disorders involving the immune mechanism)	16 (1.6)	25 (1.6)
E00–E90	(Endocrine, nutritional, and metabolic diseases)	29 (3.0)	47 (2.9)
F00–F99	(Mental, behavioral, and neurodevelopmental disorders)	18 (1.8)	37 (2.3)
G00–G99	(Diseases of the nervous system)	15 (1.5)	30 (1.9)
H00–H95	(Diseases of the eye and adnexa | Diseases of the ear and mastoid process)	3 (0.3)	6 (0.4)
I00–I99	(Diseases of the circulatory system)	70 (7.2)	124 (7.7)
J00–J99	(Diseases of the respiratory system)	72 (7.4)	107 (6.6)
K00–K93	(Diseases of the digestive system)	67 (6.9)	111 (6.9)
L00–L99	(Diseases of the skin and subcutaneous tissue)	9 (0.9)	16 (1.0)
M00–M99	(Diseases of the musculoskeletal system and connective tissue)	80 (8.2)	130 (8.1)
N00–N99	(Diseases of the genitourinary system)	30 (3.1)	48 (3.0)
Q00–R99	(Congenital malformations, deformations, and chromosomal abnormalities | Symptoms, signs, and abnormal clinical laboratory findings, not elsewhere classified)	96 (9.8)	149 (9.2)
S00–T98	(Injury, poisoning, and certain other consequences of external causes)	258 (26)	408 (25)
Z00–Z99	(Factors influencing health status and contact with health services)	147 (15)	267 (17)

### All-cause mortality

The 30-day mortality for shoulder patients was 0.61% compared with 0.28% for the matched cohort, with an IR of mortality for shoulder patients of 20.2 per 100,000 person-days compared with 9.4 per 100,000 person-days in the matched cohort, corresponding to an IRR of 2.2 (95% confidence interval [CI] 1.7–2.7). Adjusting for age, sex, and Charlson Comorbidity Index showed similar results. Mortality rates varied significantly by surgical indication, as patients with fractures had the highest mortality rate with an adjusted IRR of 3.5 (CI 2.7–4.6). Patients treated with a shoulder replacement due to osteoarthritis or cuff tear arthropathy had lower mortality rates compared with the matched cohort. Although IRs were lower at 90 days and 1 year, the associations remained consistent (see Table 3).

## Discussion

The aim of our study was to compare the IRs of SAEs, readmissions, and all-cause mortality at 30 and 90 days following shoulder replacement with those of a matched cohort. We found that SAE rates were higher across all surgical indications compared with the matched cohort, with fracture patients showing the highest. Readmission rates were highest for patients with “other” and fracture indications. The highest mortality rate was among patients with fractures while patients operated due to OA had lower mortality compared with a matched cohort.

Reported SAE rates after shoulder replacement range from 2% to 37% with an average of approximately 12% [5]. Our rates are lower but fall within this range. Higher rates of SAEs such as infections, kidney injury, and embolism may relate to surgical stress, immobility, and perioperative care. Variability across studies likely reflects differences in populations, follow-up periods, data collection, and SAE definitions [5].

Our findings of SEAs in this nationwide cohort corresponds with those found in previous studies [5-8]. Craig et al. [7] reported a 3.5% risk of SAEs at 30 days and 4.6% at 90 days for patients aged 50 and older undergoing elective surgery. [5-7,9,14-16]. Readmission rates after shoulder replacement vary across studies from 1.7% to 7.3% within 30 to 90 days [6,9,10]. In our study, the readmission rate was a little higher than previously reported rates [6,9,16]. This discrepancy may be due to our inclusion of all diagnostic codes without specifying codes for readmission. The most common reason for readmission was “injury, poisoning, and certain other consequences of external causes,” which may not be related to the surgical procedure itself.

Consistent with previous studies, we found higher mortality rates among fracture patients and lower rates among those undergoing elective surgery for osteoarthritis or rotator cuff tear arthropathy [4,5,7], suggesting that elective patients represent a generally healthier subgroup. Reported mortality ranges from 0.5% to 1.5% within 30 to 90 days, with fracture patients having higher rates at 30 days compared with the general population [4,5]. A review reported an overall 0.1% mortality rate, with individual studies ranging from 0.1–1.3% within 30–90 days [5]. Our higher rates (0.6–1.4%) likely reflect the inclusion of fracture patients, as some reviewed studies focused only on elective cases [5]. We found that fracture patients had the highest mortality (3.0–6.1%) compared with the matched cohort. Rates for proximal humeral fractures were lower than those for hip fractures (10.1–26.6%) [17], possibly due to selection bias, as frailer patients may be treated non-surgically. Nevertheless, the elevated mortality highlights a need for improved care, similar to advances in hip fracture management. While hip fractures may benefit from orthogeriatric models, shoulder fractures, despite affecting similarly frail patients, have received less attention [18].

### Limitations

There is a risk of changes in coding practices over time, variation from institution to institution in coding practices, and coding errors [19]. Implant types, perioperative care, and patient characteristics may have evolved over the study period, potentially influencing outcomes. There is a possible risk of misclassification of some diseases and conditions in the Charlson Comorbidity Index and the identification of SAEs. As not all conditions require inpatient treatment and may be managed by general practitioners, the Charlson Comorbidity Index and the number of SAEs are likely to be underestimated. The positive predictive value of the diagnosis codes included in the Charlson Comorbidity Index was high in the Danish National Patient Registry [20]. Despite statistical adjustments, the observed associations may still be influenced by unmeasured factors such as frailty. The Danish National Patient Registry has high completeness but carries a risk of information bias, as codes are not mutually exclusive [12]. Additionally, this study reports SAEs only for inpatients. The positive predictive value for the SAEs applied in this study have previously been investigated in the Danish National Patient Registry and ranges from 54% to 100% [20-25] with all SAEs having a moderate to high positive predictive value.

A potential limitation of this study is that the Danish Shoulder Arthroplasty Registry might not capture all shoulder replacements in Denmark, as a recent study found that approximately 6% of procedures may be missing [11]. In the early years of the registry, a broad range of surgical indications were accepted, which could have led to misclassification. To address this, we applied a standardized hierarchical approach to consistently classify the primary indication for all patients.

### Conclusion

We found that all surgical indications were associated with higher rates of SAEs and readmissions. Fracture patients had the highest mortality rate, while patients undergoing shoulder replacement for osteoarthritis or rotator cuff tear arthropathy had lower mortality compared with a matched cohort.

*In perspective,* these findings underscore the importance of informing patients about risks and incorporating them into shared decision-making when choosing between non-surgical and surgical treatment.

## Notes

The authors would like to thank the staff at hospital departments for their contribution to data in the Danish National Patient Registry and for caring for shoulder replacement patients. The would also like to thank the Danish Shoulder Arthroplasty Registry for the committee’s work.

Handling co-editor: Taco Gosens

*Acta* thanks Roger John Emery, Bart G Pijls, and Michael Schneider for help with peer review of this manuscript.
